# Basal Ganglia Ischemic Stroke as Sentinel Sign for Pediatric Tuberculous Meningitis in an Immunocompetent Child: A Case Report

**DOI:** 10.3390/pediatric18020044

**Published:** 2026-03-18

**Authors:** Albina Ponosheci Biçaku, Kurtesh Sherifi, Ardian Biçaku, Sadije Namani

**Affiliations:** 1 Department of Infectious Diseases, Faculty of Medicine, University of Prishtina, Str. Bulevardi i Dëshmorëve, 10000 Prishtina, Kosovo; albina.ponosheci@uni-pr.edu (A.P.B.); sadije.namani@uni-pr.edu (S.N.); 2 Clinic of Infectious Diseases, University Clinical Center of Kosovo, Lagjia Spitalit, 10000 Prishtina, Kosovo; 3 Faculty of Agriculture and Veterinary, University of Prishtina, Str. Bill Clinton, 10000 Prishtina, Kosovo; 4 Clinic of Radiology, University Clinical Center of Kosovo, Lagjia Spitalit, 10000 Prishtina, Kosovo; ardianbicaku@hotmail.com

**Keywords:** pediatric tuberculous meningitis, child, MRI, CSF, neurological sequelae

## Abstract

Background: Tuberculous meningitis (TBM) is the most severe manifestation of tuberculosis in children, with high mortality rates and long-term neurological sequelae. Early diagnosis is challenging due to its nonspecific symptoms and insidious onset. Case Presentation: An 8-year-old previously healthy male, fully vaccinated, presented with a two-week history of fever, headache, vomiting, and abdominal pain. Cerebrospinal fluid (CSF) analysis revealed lymphocytic pleocytosis, elevated protein, and low glucose levels, while multiplex polymerase chain reaction (PCR) testing for bacteria and viruses yielded negative results. Brain computed tomography (CT) revealed mild ventricular dilation and pansinusitis. Empirical antibacterial and antiviral therapy were initiated; however, the patient subsequently experienced neurological deterioration, including cranial nerve deficits and hemiparesis. Brain magnetic resonance imaging (MRI) demonstrated acute infarctions of the basal ganglia, raising suspicion for TBM. Repeated CSF sampling and Xpert MTB/RIF assay confirmed infection with *Mycobacterium tuberculosis*. Anti-tuberculosis treatment was initiated in combination with adjunctive corticosteroids, anticonvulsant and anticoagulant therapies, and supportive care, including neurosurgical intervention for hydrocephalus. After 16 months of treatment, the patient showed clinical improvement but sustained left-sided hemiparesis, visual impairment, and cognitive deficits. Conclusions: This case highlights the diagnostic challenges of pediatric TBM in immunocompetent and *Bacillus Calmette–Guérin* (BCG)-vaccinated children, particularly in the presence of initially negative microbiological findings. It emphasizes the importance of maintaining a high index of clinical suspicion and the crucial supportive role of neuroimaging findings, as well as the earlier initiation of empirical TB therapy especially when epidemiological plausibility exists. Early recognition and intervention remain critical to reducing morbidity and mortality associated with this devastating disease.

## 1. Introduction

Tuberculous meningitis (TBM) is the most severe and disabling manifestation of tuberculosis and remains associated with high mortality and long-term neurological sequelae despite appropriate therapy [1]. Although TBM accounts for approximately 1% of all tuberculosis cases and about 5% of extrapulmonary tuberculosis, it carries a disproportionate burden of morbidity and mortality, particularly among young children and immunocompromised individuals [2,3]. Children are especially vulnerable due to rapid disease progression and frequent delays in diagnosis [3]. Diagnosing TBM is more challenging than diagnosing other forms of bacterial meningitis. Unlike classical bacterial meningitis, TBM rarely presents abruptly and typically follows an insidious course, characterized by prolonged fever, headache, vomiting, and malaise, progressing to focal neurological deficits, seizures, altered consciousness, coma, and ultimately death, if left untreated [3,4]. Diagnostic delay is a critical determinant of the outcome, and untreated pediatric TBM is uniformly fatal [1,3]. Cerebrospinal fluid (CSF) analysis typically demonstrates lymphocytic pleocytosis, elevated protein concentrations, and reduced glucose levels; however, these findings are nonspecific and overlap with other causes of acute and chronic meningitis [1,3]. Microbiological confirmation remains challenging because conventional CSF cultures for *Mycobacterium tuberculosis* complex (MTPC) may require 2–6 weeks for growth [3,5]. The Xpert MTB/RIF assay performed on CSF is a valuable diagnostic tool in suspected TBM, enabling rapid detection of the disease; nevertheless, a negative result does not exclude the diagnosis, particularly in children [1,5,6]. Neuroimaging plays a crucial supportive role in the diagnosis of tuberculous meningitis and in the detection of associated complications. Magnetic resonance imaging (MRI) typically demonstrates basal leptomeningeal enhancement, hydrocephalus, and cerebral infarctions, most commonly involving the basal ganglia as a consequence of tuberculous vasculitis [1]. Ischemic stroke is a well-recognized serious complication of TBM and is associated with poor neurological outcomes [1,7]. Treatment of pediatric TBM consists of an intensive phase with isoniazid, rifampicin, pyrazinamide, and ethambutol for two months, followed by a continuation phase with isoniazid and rifampicin for at least ten months, in combination with adjunctive corticosteroids to reduce mortality and inflammatory complications [1,8]. Despite appropriate diagnosis and treatment, mortality remains high, with approximately 20% of affected children dying, and more than half of survivors developing permanent neurological sequelae, including motor, cognitive, visual, and developmental impairments [1,3]. In this report, we present the case of TBM in a previously healthy, fully vaccinated child, complicated by acute ischemic stroke and hydrocephalus, underscoring the diagnostic challenges of pediatric TBM and highlighting the importance of early clinical suspicion and repeated microbiological testing.

## 2. Case Presentation

A previously healthy 8-year-old boy (body weight 29 kg) was admitted to our institution with a two-week history of persistent fever, headache, vomiting, and abdominal pain. Initial outpatient management with symptomatic therapy and oral antibiotics failed to improve his condition, and the child remained febrile, fatigued, and progressively unwell. Due to clinical deterioration, he was referred to and hospitalized at the Clinic of Infectious Diseases for further evaluation and treatment. On admission, the patient was conscious but demonstrated neck rigidity, positive meningeal signs, and mild confusion. He was fully immunized according to the mandatory childhood vaccination schedule, including *Bacillus Calmette*–*Guérin* (BCG) vaccine, and had no personal or family history of infectious or hereditary diseases, including tuberculosis.

**Figure 1:**
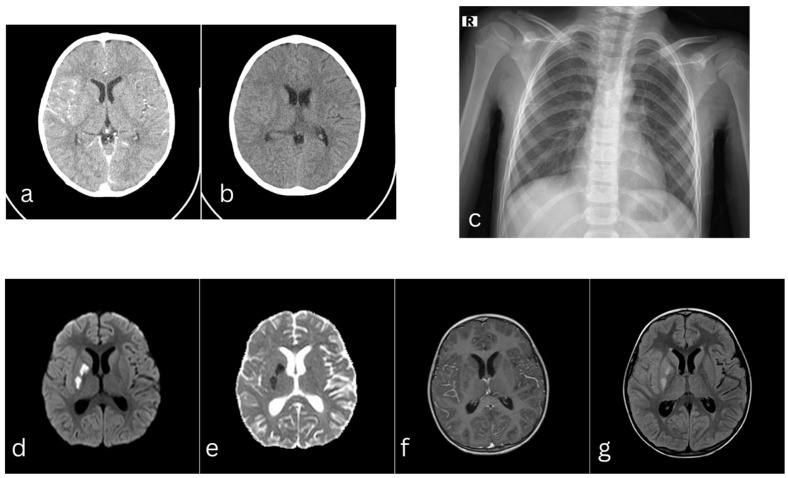
Brain CT scan with contrast (**a**), and non-contrast (**b**) on the day of admission, showing mild dilation of the ventricular system without intraventricular pathological content, vascular hypercongestion along the right temporal cerebral gyruses. Chest X-ray (**c**) shows no significant abnormalities. Brain MRI on the fifth day of admission, DWI/ADC axial scans (**d**,**e**), depict focal lesions with restricted diffusion in the right nucleocapsular region consistent with ischemic lesions. T1 axial scan with contrast (**f**) demonstrated absence of enhancement, and FLAIR axial scan (**g**) hyperintensity in the right nucleocapsular lesions.

An emergent non-contrast and contrast brain computed tomography (CT) scan demonstrated mild dilation of the ventricular system without intraventricular pathological content, vascular hypercongestion along the right temporal cerebral gyruses, and pansinusitis (Figure 1a,b). Lumbar puncture performed on the day of admission revealed clear and colorless cerebrospinal fluid (CSF) with pleocytosis of 181 cells/mm^3^ (80% mononuclear cells, 20% polymorphonuclear cells), reduced CSF glucose (1.8 mmol/L; simultaneous blood glucose 5.74 mmol/L), and elevated protein concentration (1.35 g/L). Chest X-ray showed no significant changes (Figure 1c).

Given the abnormal CSF findings and the patient’s severe clinical presentation, empirical treatment for bacterial and viral meningitis was initiated with intravenous ceftriaxone, vancomycin, dexamethasone, acyclovir, pantoprazole, and 20% mannitol.

Multiplex real-time polymerase chain reaction (rT-PCR) testing of the CSF for common bacterial pathogens (Neisseria meningitidis, Haemophilus influenzae, Streptococcus pneumoniae, Streptococcus agalactiae, Listeria monocytogenes, and Escherichia coli) and viral pathogens (including cytomegalovirus, herpesviruses, Epstein–Barr virus, varicella-zoster virus, adenovirus, parvovirus B19, enteroviruses, and mumps virus) yielded negative results.

Despite therapy, no clinical improvement was observed. On the fourth day of hospitalization, the patient developed altered consciousness, characterized by confusion, absence of verbal response, spontaneous eye opening without purposeful interaction, and mydriatic, isochoric pupils with poor light reactivity. A second lumbar puncture demonstrated persistent pleocytosis (69 cells/mm^3^, 100% mononuclear cells), CSF glucose of 2.58 mmol/L (blood glucose 6.8 mmol/L), and protein concentration of 1.13 g/L. GeneXpert MTB/RIF testing of CSF remained negative at that time.

On the fifth day of hospitalization, the patient’s neurological status deteriorated further, with the onset of right-sided ptosis, and left-sided hemiparesis. Brain MRI, including contrast-enhanced sequences, revealed acute bifocal ischemic infarctions involving the right basal ganglia (Figure 1d–g). Based on these findings, a diagnosis of acute ischemic stroke was established, and the patient was transferred to the pediatric intensive care unit (PICU).

Given the involvement of the basal ganglia and the patient’s progressive neurological deterioration, TBM was suspected, and antituberculosis therapy with rifampicin, isoniazid, pyrazinamide, and ethambutol was initiated in accordance with established protocols. The clinical course was complicated by generalized seizures and episodes of oxygen desaturation, necessitating endotracheal intubation and mechanical ventilation. Broad-spectrum antimicrobial therapy, antituberculosis drugs, corticosteroids, antiedematous agents, anticoagulant drugs and anticonvulsant agents were continued.

A repeat brain CT scan demonstrated hypodense lesions in the basal ganglia, suggesting ischemic lesions. A subsequent lumbar puncture performed on the eighth day of admission, using a larger CSF volume, yielded a positive GeneXpert MTB/RIF result, detecting *Mycobacterium tuberculosis* in low quantity, sensitive to rifampicin. Interferon-gamma release assay (QuantiFERON-TB Gold) was also positive.

Subsequently, the patient’s vital parameters stabilized, seizures were controlled, and antituberculosis therapy was well tolerated via nasogastric tube. On the 16th day of PICU admission, a tracheostomy was performed to facilitate prolonged mechanical ventilation. Follow-up brain MRI three weeks later prompted a neurosurgical recommendation for external ventricular drainage; however, the procedure was declined by the patient’s parents.

During hospitalization, ventilator-associated infections were documented, including *Klebsiella pneumoniae* and later *Stenotrophomonas maltophilia*, which were managed with targeted antimicrobial therapy based on antibiogram results.

**Figure 2:**
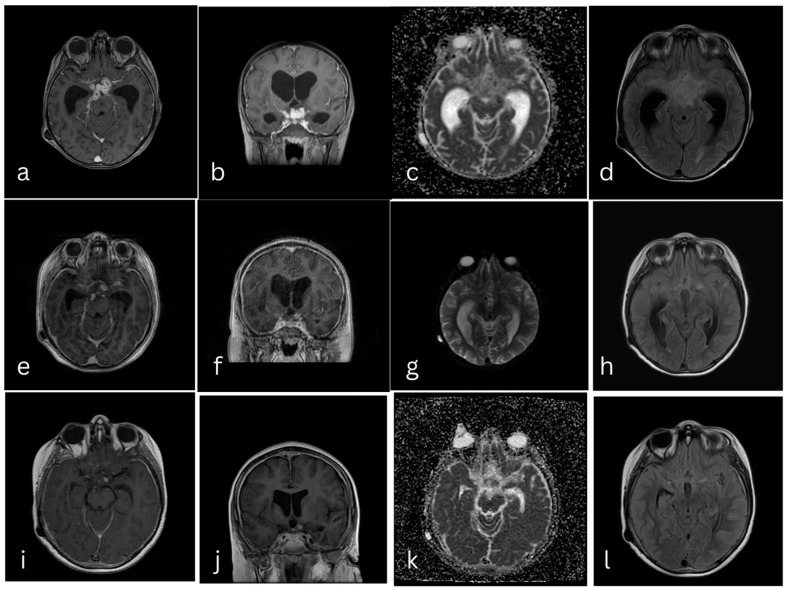
Brain MRI, T1 axial with contrast (**a**), T1 coronal with contrast (**b**), ADC axial (**c**), and FLAIR axial (**d**) after 4 months after admission, showing nodular and thickened leptomeningeal enhancement in the suprasellar region. Brain MRI 7 months after admission, T1 axial with contrast (**e**), T1 coronal with contrast (**f**), ADC axial (**g**), and FLAIR axial (**h**), showing gliosis in the nucleocapsular region. Brain MRI 12 months after admission, T1 axial with contrast (**i**), T1 coronal with contrast (**j**), ADC axial (**k**), and FLAIR axial (**l**), showing a left nodular enhancement in the suprasellar region and right hippocampal volumetric reduction.

The tracheostomy was removed after three weeks, and the patient resumed spontaneous breathing. He remained hemodynamically stable, with normal infectious parameters. Two months after admission, he underwent neurosurgical placement of a ventriculoperitoneal (VP) shunt. Antituberculosis therapy (with rifampicin and isoniazid) and anticonvulsant treatment were continued. Follow-up brain MRI was performed four months after admission, demonstrating thick and nodular leptomeningeal enhancement in the suprasellar region (Figure 2a–d), a repeat MRI at seven months, showed gliosis in the right nucleocapsular region (Figure 2e–h).

Brain MRI was repeated at 12 months during ongoing antituberculosis therapy, and demonstrated a tuberculoma in the left suprasellar region, as well as right hippocampal volumetric reduction (Figure 2i–l).

## 3. Discussion

Tuberculous meningitis (TBM) remains the most severe manifestation of tuberculosis in children and is associated with high mortality and long-term neurological disability, despite advances in diagnostic techniques and standardized treatment protocols [1,5,8]. This case highlights several well-recognized challenges in pediatric TBM, including its subacute and nonspecific clinical presentation, initial negative microbiological testing, early cerebrovascular complications, and the necessity for repeated cerebrospinal fluid (CSF) analysis to establish the diagnosis.

A previous study reported that in the decade preceding the 1999 war in Kosovo, approximately 40 cases of TBM per year were treated at the Infectious Diseases Clinic in Prishtina [9,10]. In the first decade after the war in Kosovo, the incidence of TBM decreased by 50% with a further decline in recent years [10,11]. Kosovo maintains mandatory BCG vaccination at birth primarily aimed at protecting infants and children from severe forms of tuberculosis (TB) such as TBM and miliary TB [8]. Consequently, TBM in children is now rare in our country [11], which made clinical decision-making in this case particularly challenging when considering TB therapy based solely on clinical signs and characteristic CSF findings. The diagnosis is therefore usually based on a combination of clinical, laboratory and neuroimaging findings [1,8]. In our case, with a classic CSF profile (lymphocytic pleocytosis, elevated protein, hypoglycorrhachia) and clinical deterioration, ischemic infarction in the basal ganglia on brain MRI served as sentinel sign for urgent initiation of the TB therapy.

The patient’s initial presentation with prolonged fever, headache, vomiting, and progressive neurological deterioration aligns with the typical subacute course of TBM described in previous studies [1,3,5]. Unlike acute bacterial meningitis, TBM often evolves insidiously, contributing to diagnostic delays and postponement of appropriate therapy, which is a key determinant of poor outcome [2,3]. In this case, the child was initially managed as having acute bacterial meningitis based on pathological CSF findings and early CT changes (pansinusitis), a scenario frequently reported in pediatric TBM [2,3]. The absence of known TB exposure, a normal chest X-ray, and prior BCG vaccination in this 8-year-old immunocompetent child further complicated early diagnosis, highlighting that none of these factors reliably exclude TBM, particularly in endemic regions [1,8].

CSF analysis revealed lymphocytic pleocytosis, elevated protein levels, and hypoglycorrhachia findings are typical but nonspecific for TBM and overlap with other causes of acute and chronic meningitis [2]. Initial Xpert MTB/RIF testing was negative, reflecting the known limitations of molecular diagnostics in TBM due to its paucibacillary nature and the small CSF volumes often obtained in children [1,2,6,12]. Meta-analyses have shown that although Xpert MTB/RIF facilitates rapid diagnosis and detection of rifampicin resistance, its sensitivity in TBM remains moderate, and a negative result does not exclude the disease [6,12]. In this case, repeated lumbar puncture with a larger CSF volume ultimately yielded microbiological confirmation, in accordance with current diagnostic recommendations [3,6,12].

Neuroimaging played a pivotal role in raising suspicion for TBM and guiding clinical management. Early CT imaging demonstrated nonspecific findings, which are common during the initial stages of TBM [1,2]. Subsequent MRI revealed infarctions in the basal ganglia, a characteristic complication of TBM resulting from inflammatory vasculitis affecting the perforating lenticulostriate arteries (LSAs) [1,2,5,8]. Pediatric studies have reported cerebral infarctions in TBM cases and have associated them with worse neurological outcomes [1,7,13,14]. MRI is superior to CT for the early detection of basal meningeal enhancement, tuberculomas, hydrocephalus, and ischemic lesions. Besides, diffusion-weighted imaging (DWI) can identify restricted diffusion (as a result of an ischemic lesion) before changes become apparent on CT [1,2]. Ischemic lesions secondary to arterial involvement and cranial neuropathies are common complications of basal TBM [15]. Studies report that cerebral ischemia occurs in 13–57% of TBM cases due to mechanisms including arteritis, vasospasm, and arterial thrombosis [16]. The angiographic pattern already described consists of narrowing of arteries at the base of the brain and narrowed small or medium-sized arteries. High-resolution vessel wall imaging (HR-VWI) is a relatively recent MRI technique that evaluates vessel wall structure, plaque characteristics, and inflammatory changes [17]; also it can reveal inflammatory wall thickening before luminal narrowing becomes apparent [15]. HR-VWI complements luminal imaging and can differentiate many potential causes of luminal narrowing. Vessel wall enhancement generally corresponds to expected patterns of vessel wall inflammation and/or increased vasa vasorum density in distinct vascular pathologies and can also detect cranial nerve enhancement. MRI with HR-VWI is a valuable tool for demonstrating TBM-related arteritis and can identify vessel wall enhancement without changes in the vessel caliber [15]. In this case, ischemic stroke of the basal ganglia preceded microbiological confirmation and prompted initiation of antituberculosis therapy, underscoring the diagnostic value of MRI in clinically deteriorating children.

Hydrocephalus developed during the course of illness and required neurosurgical intervention, representing another common and serious complication of TBM [1,2,3]. The patient received antitubercular therapy for 16 months, comprising a four-drug intensive phase of two months followed by an extended continuation phase, alongside adjunctive corticosteroids. Corticosteroids have been shown to reduce mortality in TBM, although their effect on long-term neurological sequelae remains limited [1,8]. Given the severity of the disease and radiological progression, antituberculosis therapy was extended beyond the standard 12 months, a strategy recommended in severe or complicated TBM cases [18,19].

Despite appropriate therapy, the patient experienced significant long-term neurological sequelae, including left hemiparesis, visual impairment, behavioral disturbance, and cognitive impairment. Such outcomes are unfortunately common; cohort studies report that more than 50–65% of children surviving TBM sustain with permanent motor, sensory, cognitive, or developmental impairments [3,5,7]. Advanced disease stage at the time of diagnosis is strongly associated with both mortality and morbidity, as a substantial portion of neurological damage occurs before effective therapy is initiated [1,3,8].

## 4. Conclusions

In conclusion, this case underscores the importance of considering tuberculous meningitis (TBM) in children of all ages who present with subacute, nonspecific symptoms of central nervous system involvement, particularly when clinical deterioration occurs despite standard antimicrobial therapy, and CSF abnormalities are consistent with the diagnosis. Early diagnosis remains challenging, and pediatric TBM continues to be associated with substantial morbidity and mortality despite appropriate treatment. Maintaining a high index of clinical suspicion, supported by characteristic neuroimaging findings, is crucial to enable the timely initiation of antituberculosis treatment.

TBM should be included in the differential diagnosis of leptomeningeal disease (particularly involving the basal cisterns) in children presenting with fever, vomiting, and cranial nerve involvement. This applies even in those who have received BCG vaccination, have lack specific X-ray findings, and have no known history of tuberculosis exposure, especially in regions with epidemiological plausibility, where earlier empirical TB therapy may be justified. Contrast-enhanced brain MRI, with or without magnetic resonance angiography, and DWI are essential for accurate diagnosis.

## Data Availability

The original contributions presented in this study are included in the article. Further inquiries can be directed to the corresponding author.
